# Serological markers for monitoring historical changes in malaria transmission intensity in a highly endemic region of Western Kenya, 1994–2009

**DOI:** 10.1186/1475-2875-13-451

**Published:** 2014-11-22

**Authors:** Jacklyn Wong, Mary J Hamel, Chris J Drakeley, Simon Kariuki, Ya Ping Shi, Altaf A Lal, Bernard L Nahlen, Peter B Bloland, Kim A Lindblade, Vincent Were, Kephas Otieno, Peter Otieno, Chris Odero, Laurence Slutsker, John M Vulule, John E Gimnig

**Affiliations:** Division of Parasitic Diseases and Malaria, Centers for Disease Control and Prevention, Atlanta, GA USA; Department of Immunology & Infection, London School of Hygiene and Tropical Medicine, London, UK; Kenya Medical Research Institute, Centre for Global Health Research, Kisumu, Kenya

**Keywords:** Malaria, *Plasmodium falciparum*, Serology, Epidemiology, Transmission intensity, Antibodies

## Abstract

**Background:**

Monitoring local malaria transmission intensity is essential for planning evidence-based control strategies and evaluating their impact over time. Anti-malarial antibodies provide information on cumulative exposure and have proven useful, in areas where transmission has dropped to low sustained levels, for retrospectively reconstructing the timing and magnitude of transmission reduction. It is unclear whether serological markers are also informative in high transmission settings, where interventions may reduce transmission, but to a level where considerable exposure continues.

**Methods:**

This study was conducted through ongoing KEMRI and CDC collaboration. Asembo, in Western Kenya, is an area where intense malaria transmission was drastically reduced during a 1997–1999 community-randomized, controlled insecticide-treated net (ITN) trial. Two approaches were taken to reconstruct malaria transmission history during the period from 1994 to 2009. First, point measurements were calculated for seroprevalence, mean antibody titre, and seroconversion rate (SCR) against three *Plasmodium falciparum* antigens (AMA-1, MSP-1_19_, and CSP) at five time points for comparison against traditional malaria indices (parasite prevalence and entomological inoculation rate). Second, within individual post-ITN years, age-stratified seroprevalence data were analysed retrospectively for an abrupt drop in SCR by fitting alternative reversible catalytic conversion models that allowed for change in SCR.

**Results:**

Generally, point measurements of seroprevalence, antibody titres and SCR produced consistent patterns indicating that a gradual but substantial drop in malaria transmission (46-70%) occurred from 1994 to 2007, followed by a marginal increase beginning in 2008 or 2009. In particular, proportionate changes in seroprevalence and SCR point estimates (relative to 1994 baseline values) for AMA-1 and CSP, but not MSP-1_19_, correlated closely with trends in parasite prevalence throughout the entire 15-year study period. However, retrospective analyses using datasets from 2007, 2008 and 2009 failed to detect any abrupt drop in transmission coinciding with the timing of the 1997–1999 ITN trial.

**Conclusions:**

In this highly endemic area, serological markers were useful for generating accurate point estimates of malaria transmission intensity, but not for retrospective analysis of historical changes. Further investigation, including exploration of different malaria antigens and/or alternative models of population seroconversion, may yield serological tools that are more informative in high transmission settings.

**Electronic supplementary material:**

The online version of this article (doi:10.1186/1475-2875-13-451) contains supplementary material, which is available to authorized users.

## Background

Measuring local malaria transmission intensity is critical for estimating the magnitude of malaria disease burden, planning evidence-based control strategies and evaluating the impact of interventions [1–3]. Moreover, malaria control programmes would benefit from monitoring long-term changes in transmission intensity in order to adapt control strategies to evolving local conditions [4]. No consensus exists, however, on which methods are most appropriate for programmatic monitoring of malaria endemicity over time, as all current tools have drawbacks [2, 4]. Hospital-based surveillance records may be readily available, but are considered unreliable as experts estimate that fewer than 20% of malaria cases and deaths are reported to the formal health system [4–6]. Inaccuracies are further compounded by presumptive diagnosis at health centres, leading to overestimation of malaria cases among those individuals seeking care [6, 7]. Entomological inoculation rate (EIR), which describes the average rate at which individuals are bitten by infective mosquitoes, is the most direct indicator of malaria transmission intensity [8, 9]. Unfortunately, measuring EIR is difficult and labour-intensive [10], and estimates can lack precision due to small-scale spatial and/or temporal heterogeneity in mosquito distributions [9, 11, 12]. In addition, EIR overestimates force of infection, the number of new infections per person per unit time, due to heterogeneous biting by mosquitoes [13, 14]. Parasite prevalence, the proportion of individuals with detectable *Plasmodium* parasites, is another common metric of malaria risk. Parasite prevalence is not a measure of incidence, however, and its relationship with force of infection is complicated by super-infection and acquired immunity [15]. Furthermore, estimates of parasite prevalence can vary widely in areas of seasonal transmission, and are influenced by the method of parasite detection, timing of measurement during the course of infection and access to anti-malarial drugs [13, 16, 17].

Anti-malarial antibodies are markers of past infection that can help to elucidate temporal trends in transmission [18, 19]. Because antibodies are longer lasting compared to patent parasitaemia and the lifespan of infective mosquitoes, serological tools are potentially more sensitive and robust than parasite prevalence or EIR. Large-scale serological surveys have proven useful in the past for examining impacts of interventions that reduce malaria parasite exposure. During the Garki Project in northern Nigeria, antibody prevalence and levels reflected recent changes in malaria exposure. Antibody responses fell abruptly during the intervention phase of the study, but rebounded soon after the intensive intervention was stopped [20]. More recently, by fitting a reversible catalytic conversion model to age-stratified seroprevalence data, investigators have estimated seroconversion rates (SCRs) that are analogous to force of infection [16, 18]. SCRs generated from several locations in Africa [16, 21, 22], Asia [23] and the Pacific [24] have shown close correlation with independent measures of transmission intensity such as malaria incidence among infants and young children, as well as averaged parasite prevalence and EIR values.

Because serological markers provide information on cumulative exposure over time [25], they are particularly well suited for evaluating long-term transmission trends [16, 18]. Data from a single cross-sectional serological survey can, in theory, be used to generate a point estimate of the current force of infection as well as analyse historic changes in exposure to infection [16, 18]. Sero-epidemiological studies from Tanzania [21], Vanuatu [24], Equatorial Guinea [22], and Swaziland [26] have confirmed that historic reductions in local malaria transmission (e.g., due to successful control strategies) can be demonstrated by a significantly lower SCR among younger cohorts born after the intervention(s). In these cases, age-seroprevalence curves exhibited a ‘break point’ signalling the timing of the change in SCR without the need for comparison against a baseline survey. To date, studies employing this method to reconstruct the timing and magnitude of transmission reduction have come from areas where transmission has dropped to low, sustained levels [21, 22, 24, 26]. It is unclear whether these serological tools are informative for reconstructing long-term malaria trends in regions of high transmission, where interventions may reduce transmission, but to a level where considerable exposure continues.

The purpose of this study was to investigate the utility of serological markers of *Plasmodium falciparum* exposure for estimating force of malaria infection and detecting temporal changes in malaria risk over an extended period in a highly endemic setting. Asembo, in Western Kenya, has historically experienced intense malaria transmission year round [27]. A community-randomized, controlled insecticide-treated net (ITN) trial conducted from 1997 to 1999 drastically reduced malaria transmission [28, 29]. Following the trial, continued high ITN coverage [30], coupled with expanded malaria interventions and health system and socio-economic improvements, led to further reductions in malaria morbidity and mortality from the late 1990s to late 2000s [31]. Despite these gains, however, malaria transmission in Asembo remains high [31].

For this study, three antigens spanning a range of immunogenicities were selected in order to identify at least one marker with sensitivity appropriate for the transmission intensities observed in Asembo. Immune responses against circumsporozoite protein (CSP) are considered short-lived, as sporozoites from each infective bite are present in the blood in small numbers and for only a short duration [32]. For this reason, anti-CSP antibodies are likely to underestimate malaria endemicity in low transmission areas [25], but be sensitive in hyper- and holo-endemic settings [33, 34]. The blood-stage antigen merozoite surface protein-1_19_ (MSP-1_19_) exhibits moderate immunogenicity and has been useful for estimating malaria endemicity across a range of low, moderate, and high transmission settings in Africa [18, 35]. Apical membrane antigen-1 (AMA-1), a blood-stage antigen known to be highly immunogenic, elicits a long-lived immune response that may cause population antibody responses to saturate at moderate transmission levels [18].

Two approaches were taken to reconstruct malaria transmission history in Asembo. First, point measurements of serological responses were calculated at five time points between 1994 and 2009, covering the period before the ITN trial to roughly a decade after the trial, for comparison against traditional malaria indices (parasite prevalence and EIR). Point measurements were also compared between villages that received ITNs in 1997 *vs* 1999 to understand if serological responses would reflect small differences in the length of population protection under ITNs. Second, within individual post-ITN years, age-seroprevalence curves were examined to look retrospectively for an abrupt drop in SCR corresponding to the timing of the ITN trial.

## Methods

This research was conducted through ongoing collaboration between the Kenya Medical Research Institute (KEMRI) and the Centers for Disease Control and Prevention (CDC).

### Study site

Asembo is located on the northern shores of Lake Victoria in Siaya County, Western Kenya. This lowland rural area (elevation 3,700 m) has served as the site of several malaria epidemiology studies and has been described in detail [27, 28, 30]. During the early 1990s, parasite prevalence in children aged < five years was 70-80% year round [36]. *Plasmodium falciparum* accounted for >95% of all clinical malaria infections [27, 36]. Predominant malaria vectors in the region included *Anopheles gambiae s.s.*, *Anopheles funestus* and *Anopheles arabiensis*, with estimated EIRs ranging from 60 to 300 infective bites per person per year [37, 38].

During the community-randomized trial carried out in Asembo from 1997 to 1999 [28], 40 of 79 villages were randomized to receive ITNs and the rest served as controls until 1999, after which they also received ITNs [29]. The two-year trial resulted in a 74% reduction in malaria force of infection [39] and >90% reduction in EIR [40] in intervention villages compared to control villages. After the trial, ITN coverage was maintained at high levels [30]. ITNs were replaced periodically, and were retreated with a pyrethroid formulation at six- to nine-month intervals by KEMRI/CDC through 2007 (J Gimnig, unpublished data). In addition, the Kenya Ministry of Health promoted scale-up of ITNs throughout this region, first through subsidized ITN distribution at antenatal clinics beginning in 2004, and later through a mass campaign distributing free long-lasting ITNs to all children aged < five years in 2006 [31].

### Entomological inoculation rates

Data on the number of blood-fed *An. gambiae s.s*., *An. arabiensis* and *An. funestus* captured, the number of houses sampled and the number of residents per house, were obtained from prior studies to estimate annual EIRs. Household mosquito collections have been conducted in Asembo since 1993, but sampling methods and trapping techniques have varied depending on the particular study in place. Bed net traps were used to collect anophelines from 1993 to 1996 [27, 32]. From 1997 to 2001, entomological collections were made by pyrethrum spray catch (PSC) [30, 40, 41]. Indoor CDC light traps were employed during the period from 2002 to 2007 [42, 43]. Since 2008, mosquito collections have again been conducted using PSCs (J Gimnig, unpublished data). For all entomological collections, anophelines were identified, sorted by sex and abdominal status and dried in individual tubes for laboratory analysis. Mosquitoes were tested for sporozoite infection by enzyme-linked immunosorbent assay (ELISA) [44] and a subset of all specimens classified as *An. gambiae s.l.* was identified to sibling species (*An. gambiae s.s.* or *An. arabiensis*) by polymerase chain reaction (PCR) [45].

### Parasite prevalence

Annual parasite prevalence for children aged one to five years was calculated using blood film data from past surveys [27, 30, 31]. During all surveys, finger-prick blood samples were collected onto slides, stained with Giemsa and then examined under a microscope for asexual *P. falciparum* parasites. Parasites were enumerated against 300 white blood cells (prior to 1996) or 500 white blood cells (since 1996). Individuals included in parasite prevalence calculations were restricted to those for whom serological specimens (see below) were also available.

### Serological specimens

Serological analyses were conducted using banked samples collected in 1994, 2000, 2007, 2008 and 2009. Samples from infants less than one year of age were excluded in all years to limit confounding of test results by maternal antibodies. Samples from 1994 were collected from a subset of 15 villages in Asembo [27], whereas samples from later years were collected across the entire Asembo area [30, 43]. In 1994 and 2000, capillary blood samples were collected into tubes and centrifuged to separate serum [27, 30], which was stored at −80°C until used for ELISAs. Immediately prior to serological testing, serum was diluted 1/200 in reconstitution buffer (phosphate buffered saline with 0.05% Tween 20 [PBS-T] containing 0.1% sodium azide).

Specimens from 2007, 2008 and 2009 were collected as dried blood spots on filter paper [46] during annual surveys conducted within the KEMRI/CDC Health and Demographic Surveillance System [31, 43, 47]. A 3-mm blood spot was punched from each filter paper and antibodies were eluted in reconstitution buffer as previously described [21]. The reconstituted blood spot solution, equivalent to a 1/200 dilution of serum, was stored at 4°C until used for ELISAs.

### Enzyme-linked immunosorbent assays

All samples (sera and reconstituted blood spots) were tested by ELISA [16, 18] for total IgG antibodies against recombinant *P. falciparum* AMA-1 (3D7; Biomedical Primate Research Center, Rijswijk, The Netherlands), MSP-1_19_ (Wellcome strain; CTK Biotech, San Diego, CA, USA) and CSP (Gennova Biopharmaceuticals, Pune, India). Briefly, recombinant AMA-1 (0.4ug/mL), MSP-1_19_ (0.2ug/mL) and CSP (1.6ug/mL) were coated onto Immunolon-4 microtiter plates (Thermo Fisher Scientific, Waltham, MA, USA). The following day, plates were washed in PBS-T, blocked with 1% milk powder (Marvel, UK) in PBS-T (PBS-T/milk), and then incubated with samples. All samples were tested in duplicate, with final serum dilutions at 1/2,000 for AMA-1, 1/1,000 for MSP-1_19_ and 1/200 for CSP. Each ELISA plate also included blank wells (PBS-T/milk only) and a positive control dilution series (pooled sera from six hyperimmune individuals from the Kisumu Blood Bank). On the third day, unbound antibodies were removed by washing in PBS-T, samples were incubated with peroxidase-conjugated rabbit anti-human IgG antibody (Dako, Carpinteria, CA, USA) (1/5,000 in PBS-T) and then plates were developed by adding o-phenylenediamine dihydrochloride (OPD) substrate (Sigma-Aldrich, St Louis, MO, USA). OPD reactions were held in the dark and stopped using 2 M H_2_SO_4_ after 11 min for AMA-1 and MSP-1_19,_ and 16 min for CSP. Optical density (OD) was read immediately at 492 nm using a plate reader (Molecular Devices SpectraMAX 340 and SoftMax Pro 5.4.1 software, Sunnyvale, CA, USA).

### Data management and statistical analysis

Duplicate OD values for each sample were imported into Microsoft Excel (Microsoft, Redmond, WA, USA), averaged and normalized against values from blank wells to adjust for background reactivity. Samples were excluded from analysis if duplicate ODs differed by over 50%. Titration curves were fit to the positive control dilution series on each plate in order to adjust sample OD values for plate-to-plate variation and allow for calculation of titres. Antibody titre was estimated using the formula: titre = dilution/[maximum OD/(OD test serum – minimum OD)-1]. Using a mixture model, cut-off values for seropositivity were determined separately for each antigen (AMA-1, MSP-1 and CSP) during each year (1994, 2000, 2007, 2008 and 2009) to avoid direct quantitative comparison of ELISA results from samples collected by different methods (serum *vs* dried blood spot). Briefly, OD values were fit by maximum likelihood as two Gaussian distributions [46] and the cut-off value was assigned as three standard deviations above the mean of the lower distribution.

To identify differences in seropositivity by sex, blood film result, or year in which the village received ITNs, logistic regression models were fit using PROC GENMOD in SAS 9.3 (SAS Institute, Cary, NC, USA). Age (continuous variable, cube root-transformed) and survey year were included to control for their effects, and models were adjusted for clustering of observations from the same household compound by incorporating an exchangeable correlation matrix.

Because data from 2000 were limited to children aged one to five years, a preliminary analysis was conducted to compare seroprevalence across the five time points specifically in this young age group. Year to year differences in the proportion of children seropositive were evaluated using Z-tests with Bonferroni correction for multiple comparisons. To calculate a measure analogous to SCR but using only data from this age group, linear regression models were fit, using median centile age as the predictor and centile seroprevalence as the outcome, to estimate the rate of increase in seroprevalence with age. This allowed for comparison of transmission intensity between years without making assumptions regarding missing older age groups. All regression lines were forced through the origin (intercept = 0) and regression coefficients were estimated in R version 3.2.0 (R Foundation for Statistical Computing, Vienna, Austria). Differences in geometric mean antibody titres (GMT) were also examined because antibody levels may respond more quickly to recent shifts in transmission than seroprevalence [22, 48]. To visualize distributions of antibody titres in more detail, reverse cumulative distribution plots were used, showing log_10_–transformed antibody titre on the X-axis and the percentage of individuals having the indicated antibody titre or higher on the Y-axis [49]. Differences in antibody titres between years were identified by Wilcoxon rank-sum test with Bonferroni correction.

To estimate population-wide SCRs in Asembo at each time point, data from all available age groups were used. Reversible catalytic conversion models were fit to age-stratified seroprevalence data using maximum likelihood in Stata 11 (StataCorp, College Station, TX, USA) [16, 18]. Seroreversion rates for each antigen were constrained for the Asembo population by first fitting a global model to aggregate data from all years. Individual models were then fit separately to datasets from each year to estimate year-specific SCRs. EIR equivalents (serology-based EIR estimates) were calculated from AMA-1 and MSP-1_19_ results using previously developed log-log calibration curves that relate SCRs to EIR values [16, 18]. EIR equivalents could not be calculated from CSP SCRs because CSP calibration curves have not yet been developed.

Datasets from years after the ITN trial concluded were examined for temporal changes in force of infection by fitting alternative reversible catalytic conversion models in which the SCR was allowed to change at a single time point. The significance of each change was evaluated using a likelihood ratio test against the initial model with no change in SCR [16, 21].

### Ethical review

Ethical approval for serological analyses was obtained from the KEMRI Ethical Review Committee and the CDC Institutional Review Board.

## Results

Demographic data for persons sampled from Asembo are summarized in Table 1. Differences in study protocols led to variation in the number and age distribution of individuals sampled during each year. Individuals from 1994 included a higher proportion of females compared to other years (χ^2^ = 34.6, p < 0.001) because pregnant females were the only persons older than 15 years surveyed that year. When pregnant females were excluded in 1994, this difference was no longer observed. The proportion of individuals with positive blood films ranged from >80% in 1994 to 36% in 2007. No year to year differences were observed in the proportion of individuals residing in villages that received ITNs at the trial’s start in 1997 *vs* at the trial’s conclusion in 1999 (χ^2^ = 3.1, p = 0.533).

**Table 1:** **Demographic characteristics for persons sampled in Asembo by year (% [n])**

		1994	2000	2007	2008	2009
		(n = 636)	(n = 629)	(n = 169)	(n = 173)	(n = 1,038)
Age (in years)	1 to 5	63.4 (403)	100 (629)	42.0 (71)	39.3 (68)	24.8 (257)
	6 to 15	17.6 (112)	0 (0)	58.0 (98)	60.7 (105)	29.2 (303)
	16 to 90	19.0 (121)	0 (0)	0 (0)	0 (0)	46.0 (478)
Sex	Female	64.3 (409)	54.1 (340)	49.4 (80)	42.1 (67)	56.9 (590)
	Male	35.7 (227)	45.9 (289)	50.6 (82)	57.9 (92)	43.1 (446)
	Not recorded	0	0	7	14	2
Blood film result^a^	Positive	80.5 (418)	54.2 (341)	36.0 (59)	42.9 (72)	36.3 (377)
	Negative	19.5 (101)	45.8 (288)	64.0 (105)	57.1 (96)	63.7 (661)
	Not recorded	117	0	5	5	0
Year village	1997^b^	47.2 (300)	47.2 (297)	42.1 (69)	43.6 (75)	48.1 (498)
received ITNs	1999^c^	52.8 (336)	52.8 (332)	57.9 (95)	56.4 (97)	51.9 (538)
	Not recorded	0	0	5	1	2
Source of samples		Asembo Bay Cohort Project^d,e^	ITN follow-up study^f^	KEMRI/CDC parasitaemia and anaemia survey^g^	KEMRI/CDC parasitaemia and anaemia survey^g^	KEMRI/CDC parasitaemia and anaemia survey^g^
Survey months		April to June	May to June	April	April	March to April

^a^Asexual *P. falciparum* parasites detected by microscopy.

^b^At the time of the survey, resided in a village that received ITNs at start of trial in 1997.

^c^At the time of the survey, resided in a village that received ITNs at end of trial in 1999.

^d^Bloland *et al.*[27].

^e^Bloland *et al.*[36].

^f^Lindblade *et al.*[30].

^g^Hamel *et al.*[31].

Across all years, the likelihood of individuals testing seropositive increased with age for all three antigens (AMA-1 OR = 6.0 [95% CI: 4.4-8.0], MSP-1_19_ OR = 2.2 [95% CI: 1.9-2.5], CSP OR = 3.0 [95% CI: 2.5-3.5], p < 0.001 for all). Seropositivity was also associated with positive blood film for AMA-1 (OR = 3.7 [95% CI: 3.0-4.5], p < 0.001) and CSP (OR = 1.8 [95% CI: 1.4-2.3], p < 0.001), but not for MSP-1_19_ (OR = 1.0 [95% CI: 0.8-1.2], p = 0.998). Females were more likely to be seropositive against MSP-1_19_ (OR = 1.4 [95% CI: 1.1-1.7], p = 0.001), whereas sex had no effect for AMA-1 (OR = 1.1 [95% CI: 0.9-1.3], p = 0.540) or CSP (OR = 1.2 [95% CI: 1.0-1.4], p = 0.133). Whether an individual resided in a village that received ITNs in 1997 or 1999 had no impact on seropositivity against any of the three antigens (AMA-1 OR = 0.9 [95% CI: 0.7, 1.1], p = 0.352; MSP-1_19_ OR = 1.0 [95% CI: 0.8-1.2], p = 0.834; CSP OR = 0.8 [95% CI: 0.7-1.1], p = 0.144).

### Serological results for young children

#### Seroprevalence

**Figure 1:**
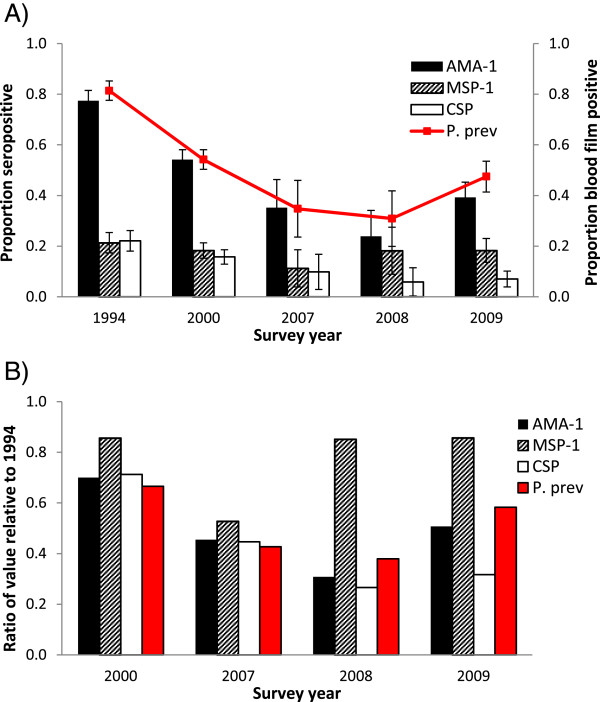
**Trends in seroprevalence compared with parasite prevalence. A)** Seroprevalence (95% CI) and parasite prevalence (95% CI) were calculated for children aged one to five years in Asembo. **B)** Ratios of seroprevalence and parasite prevalence relative to baseline values from 1994.

Figure 1A shows seroprevalence among young children compared with parasite prevalence at each time point. AMA-1 exhibited the highest seroprevalence of the three antigens tested; within each year, AMA-1 seroprevalence was typically two- to four-fold greater than that for MSP-1_19_ or CSP. AMA-1 seroprevalence fell between 1994 and 2000 (p < 0.001), and then again between 2000 and 2007 (p = 0.002). Seroprevalence against MSP-1_19_ and CSP also decreased steadily from 1994 to 2007, but the year-to-year differences were not statistically significant (p > 0.013 for all [cut-off with Bonferroni correction]). This trend of falling seroprevalence began to reverse in 2008 for MSP-1_19_ and in 2009 for AMA-1 and CSP, but none of these subsequent changes were statistically significant (p > 0.013 for all). Across the entire study period, AMA-1 seroprevalence values corresponded very closely to parasite prevalence values. Furthermore, proportionate changes in both AMA-1 and CSP seroprevalence (relative to baseline values from 1994) closely mirrored the pattern observed for parasite prevalence (Figure 1B). Proportionate changes in MSP-1_19_ seroprevalence did not follow the pattern observed for parasite prevalence, particularly in 2008 and 2009.

**Figure 2:**
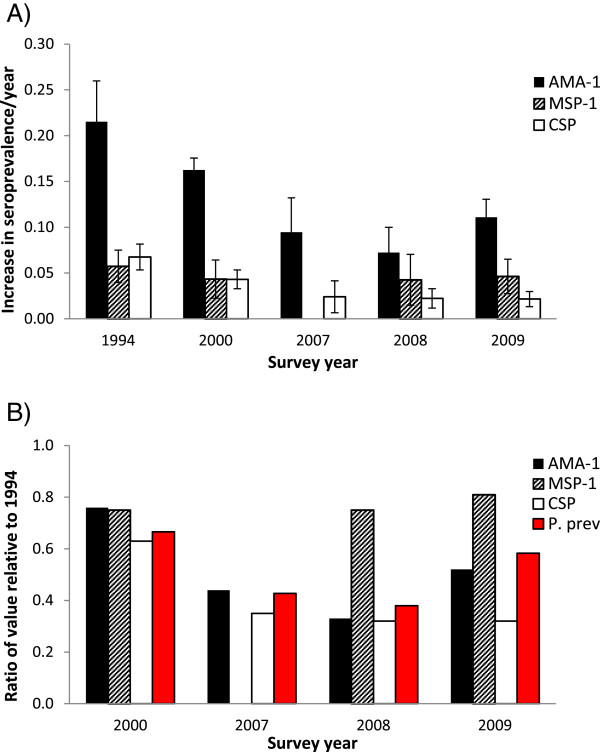
**Rate of increase in seroprevalence with age. A)** Linear regression coefficients (95% CI) describing the increase in seroprevalence per year of age among children aged one to five years in Asembo. Regression coefficients were calculated to approximate seroconversion rates using only data from young children. **B)** Ratios of regression coefficients and parasite prevalence relative to baseline values from 1994.

To approximate seroconversion rates using only data from young children, linear regression coefficients (βs) were calculated to describe the increase in seroprevalence per year of age (Figure 2A). Overall, βs for each antigen exhibited similar patterns as seen for seroprevalence (Figure 1A). Proportionate changes in AMA-1 and CSP βs were similar to those observed for parasite prevalence, whereas proportionate changes in MSP-1_19_ βs were not (Figure 2B). Within each year, no statistically significant difference in β was observed between individuals residing in villages that received ITNs in 1997 *vs* 1999 (Additional file 1).

### Antibody levels

**Figure 3:**
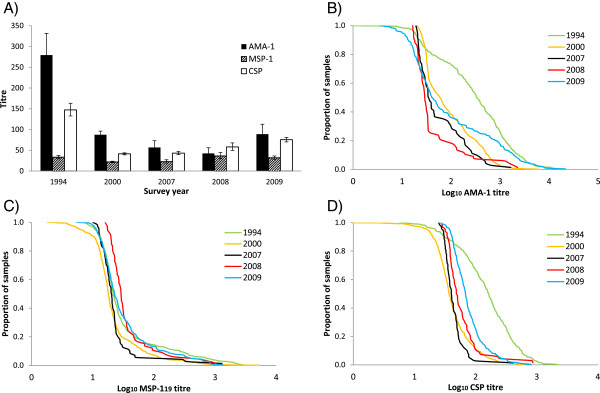
**Trends in antibody titres. A)** Geometric mean titres (95% CI) for children one to five years of age. Reverse cumulative distribution plots for: **B)** AMA-1, **C)** MSP-1_19_ and **D)** CSP. Each plot shows antibody titres (log_10_-transformed) on the X-axis and the proportion of individuals exhibiting that titre or higher on the Y-axis.

Geometric mean antibody titres for children one to five years of age are displayed in Figure 3A. Reverse cumulative distribution plots, showing the range of observed antibody titres on the X-axis and the proportion of individuals exhibiting each titre or higher on the Y-axis, are presented in Figures 3B-D. The magnitude of year-to-year change in antibody titres was greatest for AMA-1. In 2000, surprisingly, antibody titres against MSP-1_19_ were higher in villages that received ITNs in 1997 (GMT = 25.4 [95% CI: 22.8-28.2]) compared with villages that received ITNs in 1999 (GMT = 19.3 [95% CI: 17.4-21.3]) (p >0.001). No differences were detected between villages receiving ITNs in 1997 *vs* 1999 for any other antigens. Antibody titres against all three antigens fell between 1994 and 2000 (p <0.001 for all antigens). For AMA-1, titres fell further in 2007 (p <0.001), exhibited no change in 2008 (p = 0.026 [cut-off with Bonferroni correction: p = 0.013]), and then subsequently increased in 2009 (p = 0.004). MSP-1_19_ titres changed little between 2000 and 2007 (p = 0.148), but increased in 2008 (p <0.001) and then remained static in 2009 (p = 0.029). CSP titres did not change from 2000 to 2007 (p = 0.128), but increased in 2008 (p <0.001) and again in 2009 (p <0.001).

### Serological results for all age groups

#### Population SCRs

**Figure 4:**
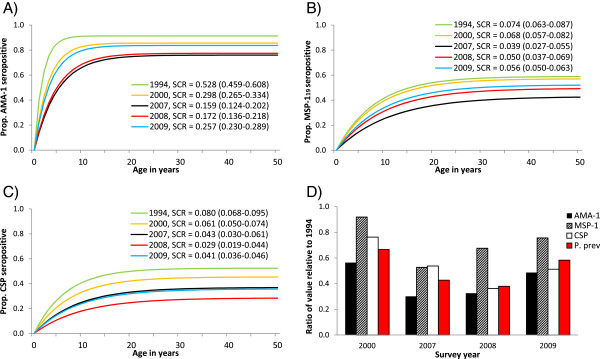
**Trends in age-seroprevalence curves and seroconversion rates (SCRs).** SCRs (95% CI) were estimated for the Asembo population by fitting reversible catalytic conversion models to data from all available age groups for: **A)** AMA-1, **B)** MSP-1_19_ and **C)** CSP. **D)** Ratios of SCR and parasite prevalence relative to baseline values from 1994.

Results from reversible catalytic conversion models fit to data from all available age groups are presented in Figure 4. Seroreversion rates in the Asembo population were fixed at 0.050 yr^−1^ for AMA-1, 0.051 yr^−1^ for MSP-1_19_ and 0.073 yr^−1^ for CSP, based on a preliminary model fit to data from all years. Consistent with the results from young children, population-wide immune responses were strongest against AMA-1. Year-specific SCRs were roughly an order of magnitude greater for AMA-1 than for MSP-1_19_ and CSP across the entire study period. SCRs for all antigens were highest in 1994, before ITN introduction. Although concerns have been raised that anti-malarial antibody responses in pregnant women may differ systematically from those of the general population [50, 51], removing pregnant women from the 1994 analysis had no significant effect on SCRs (Additional file 2). All subsequent comparisons using the 1994 dataset include samples from pregnant women.

No statistical differences in SCRs were observed for individuals living in villages that received ITNs in 1997 *vs* 1999 (Additional file 3), so within each year, data from all villages were combined for analysis. For AMA-1, SCR fell dramatically between 1994 and 2000, and again between 2000 and 2007 (Figure 4A). AMA-1 SCR remained unchanged in 2008 and then increased significantly in 2009. For MSP-1_19_, no change was observed between 1994 and 2000, but SCR fell significantly between 2000 and 2007 (Figure 4B). In 2008 and 2009, MSP-1_19_ SCR rose slightly, but this trend was not statistically significant. SCRs for CSP decreased gradually between each time point from 1994 to 2008, before increasing again in 2009 (Figure 4C), but none of these changes were statistically significant. Proportionate changes in AMA-1 and CSP SCRs (relative to baseline values from 1994) correlated closely with the pattern observed for parasite prevalence (Figure 4D). Proportionate changes in MSP-1_19_ SCR, on the other hand, were high compared to parasite prevalence in 2000, 2008, and 2009.

### EIR equivalents

**Figure 5:**
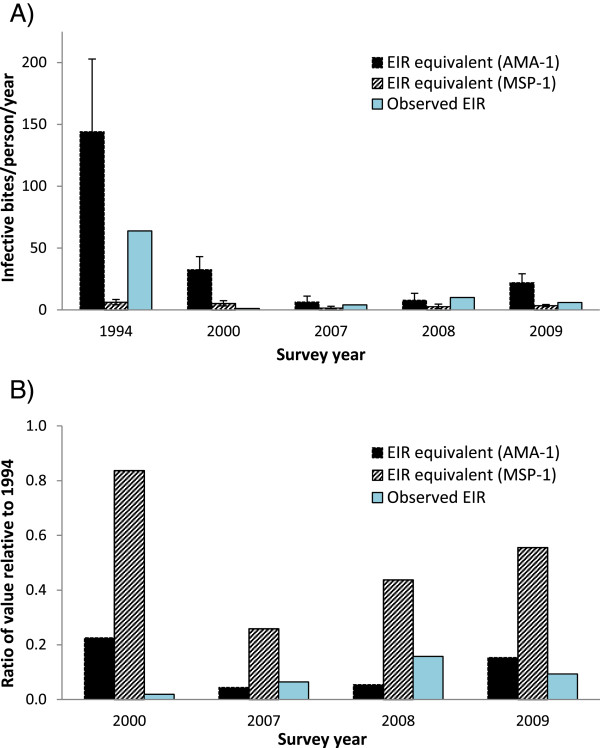
**Entomological inoculation rates (EIRs) and EIR equivalents for Asembo. A)** Observed EIRs are based on previously collected entomological data. EIR equivalents (95% CI) were estimated using AMA-1 and MSP-1_19_ SCRs and previously developed log-log calibration curves. **B)** Ratios of EIRs and EIR equivalents relative to baseline values from 1994.

Figure 5A shows EIR equivalents estimated from AMA-1 and MSP-1_19_ SCRs compared with EIR values previously measured in Asembo at the five time points [30] (J Gimnig, unpublished data). EIR equivalents based on AMA-1 showed greater variability over the study period compared to those based on MSP-1_19_. Neither antigen, however, produced EIR equivalents that closely tracked observed EIRs, or proportionate changes in EIR values, over the study period (Figure 5B).

### Changes in SCR

Because only young children, one to five years of age, were surveyed in 2000, this limited dataset could not be reliably assessed for temporal changes in SCR. For the remaining post-ITN trial datasets (2007, 2008 and 2009), none of the age-seroprevalence curves exhibited a break point suggestive of a past shift in SCR. For each antigen during each year, the initial model with only a single SCR provided a better fit than any alternative model allowing for a change in SCR.

## Discussion

Serological markers of *P. falciparum* exposure were useful for monitoring long-term changes in malaria transmission intensity in this highly endemic region. Patterns in point measurements of seroprevalence, antibody titres and SCRs all indicate that a gradual but substantial drop in malaria transmission (46-70%) occurred in Asembo from 1994 to 2007, followed by a marginal increase beginning in 2008 or 2009. This is consistent with previous findings based on child mortality, parasite prevalence [31] and EIR (J Gimnig, unpublished data). In particular, proportionate changes in antibody responses, relative to 1994 baseline values, for AMA-1 and CSP (but not MSP-1_19_) corresponded closely with trends in parasite prevalence throughout the 15-year study period.

AMA-1 is known to be highly immunogenic [18]; it was expected that AMA-1 immunological responses would saturate in the Asembo population, causing seroprevalence and SCR to remain static and therefore, uninformative. This was not the case, as AMA-1 seroprevalence and SCR varied three-fold between 1994 and 2008. Furthermore, proportionate changes for both AMA-1 seroprevalence and SCR exhibited a straightforward relationship with changes in parasite prevalence over the entire study period. As anticipated in this highly endemic region, CSP was also found to be a sensitive marker for detecting shifts in transmission; proportionate changes in CSP seroprevalence and SCR generally corresponded well with changing patterns in parasite prevalence. Despite having been informative across a range of malaria transmission settings throughout Africa [18, 35], MSP-1_19_ performed poorly in Asembo; proportionate changes in MSP-1_19_ seroprevalence and SCR overestimated changes in parasite prevalence during 2000, 2008 and 2009.

Examination of antibody titres also revealed an unexpected outcome for MSP-1_19._ In 2000 (immediately after the conclusion of the ITN trial), young children from villages that received ITNs in 1997 had significantly higher MSP-1_19_ antibody levels compared with those from villages that received ITNs in 1999. Previous investigations on short-term immunologic responses in very young children in Asembo also reported counter-intuitive results for MSP-1_19_. During a 1992–1994 cohort study following children from birth to 2.5 years, Singer *et al.*[32] found an inverse relationship between malaria exposure (based on EIR and incidence of parasitaemia) and MSP-1_19_ antibody levels. In 1998, Kariuki *et al.*[52] reported that among children under three years of age, seroprevalence against MSP-1_19_ was higher in intervention villages compared with control villages 14 months after the ITN trial’s start. At 22 months after the start of the trial, however, this pattern was no longer observed. Results from the current study, together with those of Singer *et al.*[32] and Kariuki *et al.*[52], indicate that MSP-1_19_ is not a reliable short- or long-term marker of changing malaria exposure in Asembo. Singer *et al.*[32] and Kariuki *et al.*[52] hypothesized that in this area of intense year-round malaria transmission, immune systems of young children may be so overwhelmed by high-density parasitaemia that reducing parasite exposure leads to a higher immune response against blood-stage antigens. Findings for AMA-1 from the present study suggest that this is not true for all blood-stage antigens, but rather an irregularity of the MSP-1_19_ response. This hypothesis of ineffective MSP-1 response may also explain why MSP-1 seroprevalence, unlike that of AMA-1 and CSP, was not correlated with positive blood film, an indicator of recent infection.

When comparing villages that received ITNs in 1997 *vs* 1999, it was anticipated that differences in serological responses would be apparent in 2000 and then gradually erode with time. Interestingly, aside from the unexpected finding of higher MSP-1_19_ antibody titres among children from villages that received ITNs earlier, no serological differences were found between the two groups in 2000 or in any other year. This finding suggests that immune challenge by malaria parasites remains high enough in this region to maintain antibodies against the three antigens examined, making it difficult to detect subtle differences in the length of population protection under ITNs using these serological markers.

The different serological outcomes analysed (seroprevalence, increase in seroprevalence with age, antibody titre, and SCR) all produced similar trends for reconstructing transmission history in Asembo. Of note, linear regression proved to be a useful analysis tool. The relationship between seroprevalence and age tends to be linear in young age groups, and linear regression allowed for calculation of an SCR proxy using only data from children aged one to five years. The fact that these linear regression coefficients exhibited the same pattern over time as SCRs estimated using data from all age groups supports the idea that, in regions of high malaria transmission intensity, immunological changes are most evident in young children. For example, during the classic Garki study in northern Nigeria, Cornille-Brogger *et al.*[20] also found that changes in antibody levels were most pronounced in young children up to four years of age. In Asembo, periodic point estimates of seroprevalence against AMA-1 and/or CSP in young children appear to be adequate for reconstructing long-term changes in malaria exposure. A major disadvantage, however, of any analysis method relying on point estimates is that baseline values plus several subsequent time point measurements are required for comparison. A more powerful analysis method, particularly in settings where baseline measurements are unavailable, involves fitting the reversible catalytic conversion model to seroprevalence data from all age groups to retrospectively detect changes within a single cross-sectional survey.

The reversible catalytic conversion model generated accurate population-wide SCR estimates, but did not detect retrospective changes associated with the 1997–1999 ITN trial within individual post-trial surveys. Because EIR, parasite prevalence and child mortality statistics from Asembo all indicated that malaria transmission declined drastically as a result of the ITN trial [30, 39, 40, 53], children born after the trial were expected to exhibit a markedly reduced SCR compared with older age groups. However, no break points indicating change in SCR with age were found within the age-seroprevalence curves from 2007, 2008 or 2009. It is possible that changes in SCR would have been most pronounced immediately after the trial’s conclusion. Unfortunately, the serological samples from 2000 were restricted to young children aged one to five years, limiting the ability to assess for shifts in SCR across different age groups during that year.

Inability to detect break points in the 2007, 2008 or 2009 age-seroprevalence curves may be due to several non-mutually exclusive factors. First, the Kenya Ministry of Health has instituted a series of national interventions over the past decade, including introduction of long-lasting ITNs, revised recommendations for more effective first-line anti-malarials [31] and implementation of intermittent preventive treatment in pregnant women [47] as well as integrated management of childhood illness [54]. These health system changes, along with socio-economic improvements, have further reduced malaria morbidity and mortality in Asembo [31]. Thus, Asembo residents have experienced a gradual decline in malaria transmission over many years, making the reductions from the ITN trial more difficult to pinpoint. Second, despite the documented reductions in malaria morbidity and mortality over the last decade, transmission intensity in Asembo remains high compared to other malaria-endemic areas. When examining data from other locations where clear break points have been documented in the age-seroprevalence curves (e.g., Tanzania [21], Vanuatu [24], Equatorial Guinea [22]), post-intervention SCRs were generally two to ten times lower than the values observed for Asembo. It is possible that transmission intensity remains too high in Asembo for the reversible catalytic conversion model, combined with these antigens, to accurately predict shifts in SCR between age groups. Third, a great deal of short- and long-term migration occurs in and out of the Lake Victoria region [43, 55, 56]. Out of this highly mobile population, it is possible that many of the individuals surveyed in 2007, 2008 or 2009 did not reside in Asembo during the ITN trial, further diminishing the ability to detect a sharp drop in malaria exposure. Fourth, serological evidence of the historic decline in transmission may be obscured in part by the recent rise in malaria incidence in Asembo since 2008. While serological data from 2007 (and potentially 2008) would not have been influenced by this problem, samples from those two years were small in number and restricted to individuals aged ≤15 years, which could have hindered the ability to detect differences in SCR between age groups. Samples from 2009 were ample and included all ages, but the rebound in malaria transmission may have obscured any differences between age groups by disproportionately increasing seroprevalence in younger individuals.

In addition to limited sample sizes and age groups for certain years, reliance on past studies that employed different protocols presented multiple challenges. For example, serological specimens from 1994 and 2000 were collected as serum, whereas specimens from 2007 to 2009 were collected as dried blood spots. To be cautious, direct quantitative comparison of ELISA results between years was minimized by calculating different seropositivity cut-off values for each year. Unfortunately, direct comparisons could not be avoided when examining antibody titres. It should be noted, however, that Corran *et al.*[46] reported 93% correlation in antibody concentrations between paired serum and blood spot samples from the field, suggesting that both types of specimens serve as adequate and comparable sources of anti-malarial antibodies.

Different methods were also employed to measure EIRs in Asembo over time. Bed net traps, PSCs and CDC light traps differ in their sensitivity for capturing malaria vectors [11, 57, 58], making it difficult to compare EIRs from one year to the next. This problem extends far beyond the present study; EIR is the most commonly used metric of malaria transmission intensity, yet is notoriously difficult to standardize [3]. The lack of congruence between EIR equivalents estimated from serological data and measured EIR values could be due to poor fit of the calibration curves to Asembo serology data, failure to standardize EIR measurement techniques, or a combination of both. In addition, the heterogeneous distribution of mosquito bites across individuals within a population is known to introduce discrepancies between EIR and SCR [14]. Despite these problems, the overall trend of a dramatic decrease in EIRs and EIR equivalents from 1994 to the mid 2000s, followed by a small rise in recent years, is consistent with serological results [31].

## Conclusions

Sero-epidemiological tools have proven valuable for quantifying long-term changes in malaria transmission in numerous different locations. From the standpoint of programmatic monitoring, serology is relatively fast and efficient; finger-prick blood samples can be quickly collected in large numbers and ELISAs are high-throughput and easily standardized [16, 46]. In highly endemic areas, however, historic reductions in malaria transmission may be difficult to detect from a single post-intervention survey using current serological tools, potentially limiting the utility of these tools for retrospective analyses. Nevertheless, in high transmission settings, serological point measures could be effective as adjunct tools used in combination with parasite prevalence, as serology provides information on cumulative incidence and is less sensitive to seasonal fluctuations. Exploration of different malaria antigens or alternative models of population seroconversion (e.g., models that take into account age- or exposure-related changes in seroreversion rates [48, 59–61]) may yield tools that are more informative for retrospective analyses in high endemicity areas. As malaria control receives increasing attention and funding, there will be growing need for tools that can accurately measure the epidemiological impact of interventions over extended periods, particularly in high transmission settings where reductions are most difficult to achieve and sustain.

## Electronic supplementary material

Additional file 1:**Increase in seroprevalence with age in villages that received ITNs in 1997** ***vs*** **1999.**(DOCX 13 KB)

Additional file 2:**Seroconversion rates (SCRs) for 1994 dataset including and excluding pregnant women.**(DOCX 14 KB)

Additional file 3:**Seroconversion rates (SCRs) for villages that received ITNs in 1997** ***vs*** **1999.**(DOCX 15 KB)
