# Supplementary material for: Serological markers for monitoring historical changes in malaria transmission intensity in a highly endemic region of Western Kenya, 1994–2009
Source: Malar J. 2014 Nov 22;13:451. doi: 10.1186/1475-2875-13-451 (PMC4258276; doi:10.1186/1475-2875-13-451)
Supplement: Supplementary file 1 — Additional file 1:Increase in seroprevalence with age in villages that received ITNs in 1997 vs 1999.(DOCX 13 KB) [file 12936_2014_3619_MOESM1_ESM.docx]

**Table S1 Increase in seroprevalence with age in villages that received ITNs in 1997 *vs* 1999**

|  | AMA-1 β  (95% CI) | | MSP-1_19_ β  (95% CI) | | CSP β  (95% CI) | |
| --- | --- | --- | --- | --- | --- | --- |
|  | Village received ITNs in 1997 | Village received ITNs in 1999 | Village received ITNs in 1997 | Village received ITNs in 1999 | Village received ITNs in 1997 | Village received ITNs in 1999 |
| 2000 | 0.154  (0.141-0.168) | 0.170  (0.149-0.190) | 0.050  (0.019-0.081) | 0.038  (0.023-0.052) | 0.035  (0.026-0.044) | 0.051  (0.037-0.064) |
| 2007 | 0.064  (0.012-0.116) | 0.127  (0.088-0.167) | 0.028  (-0.004-0.060) | 0.020  (0.001-0.039) | 0.015  (-0.002- 0.032) | 0.032  (-0.001-0.066) |
| 2008 | 0.058  (0.031-0.085) | 0.108  (0.056-0.160) | 0.040  (-0.010-0.090) | 0.046  (0.029-0.064) | 0.036  (0.016-0.056) | 0.00 |
| 2009 | 0.110  (0.088-0.132) | 0.110  (0.088-0.132) | 0.045  (0.027-0.063) | 0.048  (0.022-0.073) | 0.012  (0.004-0.021) | 0.032  (0.014-0.050) |

Linear regression coefficients (βs) describing the increase in seroprevalence per year of age among children aged one to five years in Asembo. At the time of each survey, children were dichotomized as residing in a village that either received ITNs in 1997 or 1999. Regression coefficients were calculated to approximate seroconversion rates using only data from young children.
