# Supplementary material for: Serological markers for monitoring historical changes in malaria transmission intensity in a highly endemic region of Western Kenya, 1994–2009
Source: Malar J. 2014 Nov 22;13:451. doi: 10.1186/1475-2875-13-451 (PMC4258276; doi:10.1186/1475-2875-13-451)
Supplement: Supplementary file 2 — Additional file 2:Seroconversion rates (SCRs) for 1994 dataset including and excluding pregnant women.(DOCX 14 KB) [file 12936_2014_3619_MOESM2_ESM.docx]

**Table S2**  **Seroconversion rates (SCRs) for 1994 dataset including and excluding pregnant women**

|  | Including pregnant women | Excluding pregnant women |
| --- | --- | --- |
| AMA-1 SCR  (95% CI) | 0.528  (0.459-0.608) | 0.581  (0.502-0.672) |
| MSP-1_19_ SCR  (95% CI) | 0.074  (0.063-0.087) | 0.077  (0.064-0.092) |
| CSP SCR  (95% CI) | 0.080  (0.068-0.095) | 0.083  (0.069-0.100) |

SCRs (95% CI) were estimated for the Asembo population by fitting reversible catalytic conversion models to data from all available age groups. In 1994, pregnant females were the only persons older than 15 years surveyed. Due to concerns that anti-malarial antibody responses in pregnant women may differ systematically from those of the general population, SCRs were calculated including and excluding pregnant women.
