# Supplementary material for: Serological markers for monitoring historical changes in malaria transmission intensity in a highly endemic region of Western Kenya, 1994–2009
Source: Malar J. 2014 Nov 22;13:451. doi: 10.1186/1475-2875-13-451 (PMC4258276; doi:10.1186/1475-2875-13-451)
Supplement: Supplementary file 3 — Additional file 3:Seroconversion rates (SCRs) for villages that received ITNs in 1997 vs 1999.(DOCX 15 KB) [file 12936_2014_3619_MOESM3_ESM.docx]

**Table S3**  **Seroconversion rates (SCRs) for villages that received ITNs in 1997 *vs* 1999**

|  | AMA-1 SCR  (95% CI) | | MSP-1_19_ SCR  (95% CI) | | CSP SCR  (95% CI) | |
| --- | --- | --- | --- | --- | --- | --- |
|  | Village received ITNs in 1997 | Village received ITNs in 1999 | Village received ITNs in 1997 | Village received ITNs in 1999 | Village received ITNs in 1997 | Village received ITNs in 1999 |
| 2000 | 0.269  (0.227-0.319) | 0.326  (0.278-0.382) | 0.086  (0.068-0.110) | 0.053  (0.031-0.070) | 0.046  (0.033-0.064) | 0.074  (0.058-0.096) |
| 2007 | 0. 130  (0.088-0.192) | 0.182  (0.131-0.251) | 0.059  (0.036-0.095) | 0.029  (0.017-0.049) | 0.043  (0.024-0.075) | 0.040  (0.025-0.065) |
| 2008 | 0.186  (0.131-0.264) | 0.162  (0.118-0.223) | 0.048  (0.030-0.078) | 0.050  (0.033-0.076) | 0.038  (0.022-0.066) | 0.022  (0.012-0.040) |
| 2009 | 0.242  (0.205-0.285) | 0.273  (0.232-0.320) | 0.053  (0.044-0.063) | 0.059  (0.051-0.070) | 0.034  (0.028-0.041) | 0.047  (0.040-0.057) |

SCRs (95% CI) were estimated for the Asembo population by fitting reversible catalytic conversion models to data from all available age groups. At the time of each survey, individuals were dichotomized as residing in a village that either received ITNs in 1997 or 1999. Seroreversion rates for the entire Asembo population were fixed at 0.050 yr^-1^ for AMA-1, 0.051 yr^-1^ for MSP-1_19_ and 0.073 yr^-1^ for CSP, based on a preliminary model fit to data from all years.
